# The dynamics of burnout among Slovenian primary school teachers over the school year in relation to their perceptions of various predictors of burnout in the school context

**DOI:** 10.3389/fpsyg.2023.1108322

**Published:** 2023-02-02

**Authors:** Neja Markelj, Marjeta Kovač, Gregor Jurak

**Affiliations:** Faculty of Sport, University of Ljubljana, Ljubljana, Slovenia

**Keywords:** dynamics of burnout, individual and environmental predictors of burnout, longitudinal, MBI-ES questionnaire, stressor, teacher burnout

## Abstract

This study examined the dynamics of teacher burnout over the course of the school year in relation to individual and environmental factors in the school context based on a three-wave panel design using an MBI-ES questionnaire and a self-constructed inventory to measure individual and environmental factors of burnout. The sample consisted of 718 teachers from 32 Slovenian primary schools; 163 of them participated in all measurements. The major limitation of this study is the high attrition rate. However, attrition analysis showed no significant differences between the initial sample and the panel group on background variables and burnout dimensions or on environmental and individual factors. Burnout was present but not pronounced among participating teachers: Emotional exhaustion was moderately high and depersonalization and personal accomplishment were low. Over the course of the school year, burnout did not increase consistently and gradually; we found only a statistically significant increase in personal accomplishment in the middle of the school year and a statistically significant greater sense of burnout at the end of the school year. As stress accumulates over time, we would expect burnout to increase. We hypothesize that participants reduced the effects of stress through various coping strategies and/or replenish their resources. We believe that the school year is not long enough for burnout to develop. The number of stressors perceived by teachers was significantly related to burnout rates. Teachers experience stress, especially in work not directly related to teaching, and from their own performance expectations. Multivariate regression analyses yielded three different but similar models of predictors of burnout that explained 25 to 50% of the variance in teacher burnout. Regardless of the instability of the models, the time and energy demands of working with students, teacher characteristics, and classroom management are the stable antecedents in the predictor models of teacher burnout.

## 1. Introduction

The burnout phenomenon was originally described among professionals working in the human services field, particularly in professional care (Maslach et al., 1996). Burnout is a serious occupational risk that results from an extensive and prolonged response to work stress in the workplace (Maslach et al., 2001; Pietarinen et al., 2021). It manifests itself in three dimensions: emotional exhaustion (EE), depersonalization (DP), and a diminished sense of personal accomplishment (PA) and occurs in individuals who work with people (Maslach et al., 1996, 2001).

Education is one of the most stressful work environments (Johnson et al., 2005; EU-OSHA, 2009; Fisher, 2011; Grant-Rankin, 2017; Worth and Van den Brande, 2019) because it involves multiple tasks (e.g., lesson preparation and classroom management) and interactions with multiple groups of people (e.g., students, colleagues, and parents; Jensen et al., 2012). The intense relational nature of classrooms means that teachers are vulnerable to emotionally draining and discouraging experiences (Maslach et al., 1996); therefore, the potential for emotional distress for teachers is high. Grant-Rankin (2017) argues that teacher burnout is already a universal phenomenon: nearly half of teachers in India are burned out (Shukla and Trivedi, 2008 in Grant-Rankin, 2017), half of South Jordanian teachers of both genders are emotionally exhausted (Alkhateeb et al., 2015 in Grant-Rankin, 2017), 15% of Swedish teachers show high rates of burnout on at least two dimensions of burnout and 4% of teachers on all (Arvidsson et al., 2016), and 83% of Slovenian primary school teachers reported a decrease in PA, 58% reported an increase in EE, and 22% reported an increase in DP (Depolli Steiner, 2014).

Prolonged exposure to psychosocial hazards leads to burnout, usually initially through EE to DP and decreased PA (Maslach and Leiter, 2016). EE is the most important and obvious manifestation of burnout (Maslach and Leiter, 2016). The authors suggest that teacher burnout begins with excessive stress working with students, parents, and other school personnel, while on the other hand, there is a lack of support at work and effective stress management skills. This leads to EE in teachers, which manifests in a lack of positive emotions and feelings such as frustration, anger, hostility, anxiety, and restlessness. Emotionally exhausted teachers begin to emotionally distance themselves in their interactions and do not engage with students as they would like. Increasing EE releases depersonalizing behaviors as a defense mechanism (increased DP). Teachers begin to underestimate their students’ problems, even though they are still professionally active and enjoy their educational accomplishments. The teacher’s disinterest in the students or his/her negativity and harshness toward the students promotes negative behaviors in the students. Thus, the teacher faces more and more failures and feels a low PA. Teachers do not feel that they have an impact on students and therefore may be disappointed in themselves or their work.

Llorens-Gumbau and Salanova-Soria (2014) suggest that the development of burnout in teachers may begin with chronic job demands or stressors, which in turn can deplete their energy resources and lead to burnout. They found that both causal and reverse causal relationships are simultaneously active in the reciprocal relationships between structural (obstacles and facilitators) and affective factors (burnout and engagement) and work self-efficacy. Kim and Burić (2020) report similar findings: Experiencing burnout (both exhaustion and disengagement) predicts teachers’ future self-efficacy levels more strongly than the other way around.

Several research findings (e.g., Hakanen et al., 2008; Schaufeli et al., 2011; Kim and Burić, 2020) suggest relative stability in burnout levels over time, suggesting that teachers will experience similar levels of burnout if no changes are made (Kim and Burić, 2020). Therefore, it is important to examine the associations between teacher burnout and other factors and constructs in order to ameliorate or even prevent the development of burnout.

In the past, many studies have examined the correlates of teacher burnout. Research suggests that burnout in teachers is influenced by subjective factors rather than objective factors. For example, a meta-analysis on teacher burnout found that gender, marital status, subject area, and educational status had very little influence on teacher burnout, although the differences were statistically significant (Yorulmaz and Altınkurt, 2018).

Previous research has also contributed to understanding the contribution of job characteristics to primary teacher burnout. In the past, researchers have consistently reported similar sources of stress for primary school teachers. For example, Thomas et al. (2003) found that time and workload pressure were ranked as the greatest source of stress, followed by parental expectations and demands, student behavior and student problems, negative community attitudes toward teaching, problems with school administration and staff, and lack of professional recognition. In Herman and Reinke (2015), reported similar sources of primary teacher stress, including (a) administrative expectations, (b) challenging colleagues, (c) time demands and limited resources, (d) diverse student needs and differentiated instruction, (e) student behaviors and attitudes, (f) lack of preparation, (g) working with parents, and (h) life stress. Stressors from all listed sources of stress are repeatedly identified as facilitators of burnout in primary school teachers. For example, high workload and pressure, work-privacy conflict, job insecurity, staff affiliation and consensus on mission, destructive social interactions, emotional demands, noise and vocal stress, classroom management, student achievement, poor cooperation, inadequate support, lack of planning and preparation, and task orientation have been found to increase the risk of burnout for teachers (Kokkinos, 2007; Stauffer and Mason, 2013; Aloe et al., 2014; Malinen and Savolainen, 2016). In turn, autonomy, supervisory support, opportunities to receive constructive feedback, and professional recognition have been shown to reduce risk (Kokkinos, 2007; Stoeber and Rennert, 2008).

Over the past decade, much of the research has focused on explaining individual characteristics of primary teachers related to burnout. For example, affectivity, novelty seeking, persistence, self-direction, workplace engagement, resilience, self-efficacy, neuroticism, conscientiousness, extraversion, burnout crossover, stress coping strategies, proactive self-regulation strategies (Kokkinos, 2007; Brudnik, 2009; Mojsa-Kaja et al., 2015; Burić et al., 2019; Meredith et al., 2020; Pietarinen et al., 2021; Shakeel et al., 2022).

Many studies have helped to understand the contribution of job characteristics and individual teacher factors to classroom stress separately; however, their joint effect has not been adequately explored. Collie and Mansfield (2022) report that for some teachers and schools, certain sources were more important than others. Thus, they emphasize the importance of considering both individual and school contexts when addressing and reducing stress in interventions.

Transactional theories have an even more complicated position on the relationship between constructs. Recall first: according to Selye (1946 in Fimian, 1982), stress is a hypothetical construct that represents a state of equilibrium between the individual responding to environmental demands and the actual environment, and stressors are events in the environment that represent a change in the usual environmental conditions and require extensive adaptive responses from the individual. Transactional theories understand stress as an internal state in which an individual’s psychological characteristics (e.g., personality traits, cognitive appraisal of the stressor, stress coping strategies, social support, previous experiences with the stressor, or current mood) moderate the relationship between the environment and the stress (Biggs et al., 2017). These can mitigate or amplify the effects of the stressor: Teachers interpretation of the situation (perception of and attitudes toward the stressor and their own resources for overcoming it) is important to the intensity and duration of the stressor (McCormick and Barnett, 2011; Parker et al., 2012). Psychological characteristics found to moderate the impact of external stressors in primary teachers include coping strategies (e.g., Herman et al., 2020), self-efficacy (e.g., Shakeel et al., 2022), teacher reflection (e.g., Košir et al., 2015), neuroticism (e.g., Goddard et al., 2006), striving and occupational commitment (e.g., Jepson and Forrest, 2006), psychological capital and flourishing (Freire et al., 2020), irrational beliefs (e.g., Bernard, 2016), and job satisfaction (e.g., Zang et al., 2022). It is also important what individuals do when they perceive high levels of stress: poor recovery experiences in the form of low leisure relaxation and non-restorative sleep partially mediated the relationship between effort-reward imbalance and lower occupational efficiency (Gluschkoff et al., 2016). In addition, Zhao et al. (2022) found that work–family conflict plays a mediating role between job stress and burnout among primary and secondary teachers. Therefore, teachers may perceive, interpret and respond to different sources of stress differently (Collie and Mansfield, 2022).

### 1.1. Research problem

Limited research has been conducted on the development of primary teacher burnout during the school year. For example, study of Capel (1991) showed no significant differences between teacher burnout at the three time points during the year, although most teachers showed the highest burnout in February. The profile analysis showed that the direction of change (if any) was not necessarily the same between the three time points. This suggests that there are some factors in the school environment that influence the development of burnout.

However, there are still many unexplained relationships between the development of primary teacher burnout on the one hand and the school environment and primary teacher characteristics on the other. Therefore, the purpose of this study is to examine the dynamics of burnout in primary teachers over the course of the school year in relation to their perceptions of various stressors in the school environment.

We hypothesize that the experience of burnout changes and intensifies during the school year. During the school year, some stressors are constantly present, while there are a number of stressors that are one-time, transient, or sporadic. Because stress accumulates over time (Taris et al., 2001), we hypothesize that perceived burnout increases during the school year.

According to transactional theories, teachers’ cognitive appraisal of stressors is an important mediator of burnout development (Depolli Steiner, 2014). Therefore, we hypothesize that the number and importance of primary teachers’ perceived stressors should correlate with perceived burnout. If individuals do not eliminate the negative effects of stress, the effects of microstressors add up (Taris et al., 2001).

A literature review by Thomas (2004) pointed to the associations between perceptions of work and feelings of overwhelm and suggested that “feeling overwhelmed at work” may be an antecedent and possible proxy for EE and DP. For example, subjective workload predicted high levels of burnout in students, while actual workload did not (Jacobs and Dodd, 2003). Thus, another aim of this study is to examine the relationship between subjective feelings of primary teacher burnout, measured primary teacher burnout, and environmental and individual factors. We hypothesize that there is a positive correlation between subjective and measured primary teacher burnout on the one hand and environmental and individual factors on the other.

## 2. Materials and methods

### 2.1. Participants and procedure

The study was conducted at three time points in a school year: 1 week before fall break (late October—T1), 1 week before winter break (mid-February—T2), and 1 week before the end of the school year (mid-June—T3).

At the beginning of the school year, a letter was sent to 45 primary schools (10% of all Slovenian primary schools) from all 12 statistical regions explaining the purpose of the survey and asking for their cooperation. Schools were sampled in two steps: (1) by region and (2) by school size and urban/rural setting. The response rate was 71.1% (32 schools): 13 large and 19 small, 10 urban and 22 rural. After obtaining consent from the school principals, one of the researchers attended staff meeting where she explained the purpose and the procedure of the study to the participants and conducted the initial assessment in the teacher conference. School coordinators conducted the other two assessments (handing out questionnaires for participants to answer individually) and returned the paper questionnaires by mail. The first data collection took up to an hour, the second up to 30 min, and the third up to 10 min.

Teachers participated in the study voluntarily. From this point on, we will address primary teachers as teachers. The total sample consisted of 718 teachers (89.3% female). Their mean age was 40.9 years, age ranged from 20 to 62 years, and mean teaching experience was 17.4 years (range of 0 to 41 years). 82.2% of the teachers had a permanent employment and 57.4% were classroom teachers. Most of the participating teachers taught at the grade level (43.9%), 31.5% at the subject level, and 16.6% at both levels. The percentage of teachers from small or large schools was similar (55%/45%), while more teachers were from rural than urban schools (62.0%/38.0%).

At T1, 614 teachers participated, at T2, 306 participated, and at T3, 321 participated (Table 1). Across all three measurements, 22.7% of teachers participated (*N* = 163). At least 360 teachers (50.1%) participated in at least two of the measurements.

**Table 1:** The number of teachers participating in three time points.

I	II	III	Teachers	
			*N*	%
Yes	Yes	Yes	163	22.7
		No	90	12.5
	No	Yes	79	11.0
		No	282	39.3
No	Yes	Yes	28	3.9
		No	25	3.5
	No	Yes	51	7.1

Results on dynamic of burnout were calculated using the final sample of 163 teachers who participated at all three time points. In the final sample, 11.0% of teachers were male and 89.0% were female. Their mean age was 40.9 years, age ranged from 25 to 58 years, and mean teaching experience was 17.5 years (range of 0 to 36 years). Compared to the total sample, more teachers (85.3%) had temporary employment and more teachers (60.1%) were classroom teachers. Most of the participating teachers taught at the grade level (42.9%), 33.7% at the subject level, and 17.8% at both levels. Similar to the total sample, more teachers were from small (59.5%) and rural schools (73.6%), although both percentages were higher.

### 2.2. Variables

This study was part of a larger research project aimed at examining the effects of individual and environmental factors, coping mechanisms, and resource replacement on the dynamics of burnout among teachers during the school year. Data for this study came from three domains: (1) demographic and job information, (2) assessment of burnout, and (3) assessment of environmental and individual factors.

*The demographic and job information domain* included questions on: gender, age, years of teaching experience, type of employment (permanent/temporary employment), school district (urban/rural), educational level, and whether or not you are a classroom teacher. Participants had to generate their own code. In case they forgot it, all listed variables were measured in all three waves to identify the questionnaires of the same person.

*Burnout* was measured using the MBI-ES questionnaire (Maslach et al., 1996). It consists of 22 items measured on a 7-point Likert scale. These items measure the frequency of experiencing the three independent dimensions of burnout: (1) EE (nine items), (2) DP (five items), and (3) PA (eight items). The individual’s degree of burnout is expressed by a high EE and a high feeling of DP and a low PA. The reliability coefficients of the subscales in the original study were as follows: *α_EE_* = 0.90, *α_DP_* = 0.79, *α_PA_* = 0.71 (Maslach et al., 1997). The three-factor structure of the Slovenian translation of MBI-ES was confirmed with principal component analysis (PCA) and the reliability of the instrument was measured with Cronbach’s alpha: *α_EE_* = 0.88, *α_DP_* = 0.84, *α_PA_* = 0.54 (Depolli Steiner, 2014). The internal consistency of the instrument was also estimated in this study at the first measurement (*N* = 614) using Cronbach’s coefficient alpha. Reliability coefficients for the subscales were as follows: *α_EE_* = 0.90, *α_DP_* = 0.68, *α_PA_* = 0.77.

An additional question (“*To what extent do you feel burned out?*”) measured subjective feelings of burnout (SFB) on a 5-point Likert scale (1—*I do not feel burned out at all*; 5—*I feel completely burned out*). SFB is highly and positively significantly correlated with EE at all three time points (0.74 ≤ *r* ≤ 0.78), positively statistically significantly correlated with DP at all three time points (0.32 ≤ *r* ≤ 0.43), and negatively significantly correlated at T2 and T3 (−0.4 ≤ *r* ≤ −0.26).

*Assessment of environmental and individual factors* was measured using a self-constructed inventory developed in an independent preliminary study (Markelj, 2008) to identify specific factors that correlate with burnout in teachers. The questionnaire was administered at T1. It consists of 42 items that were rated on a 5-point Likert scale (1—*I strongly disagree*; 5—*I strongly agree*). PCA of the total sample (*N* = 614) yielded 13 factors (Kaiser-Guttman criterion, scree plot analysis): teacher characteristics (e.g., *I am steadfast*; *I am positively life-oriented*), time and energy demands of working with students (e.g., *I find it difficult to work with a heterogeneous class*), student learning characteristics (e.g., *my students have poor study habits*), administration and job responsibilities (e.g., *I have a lot of administrative work*), ambition (e.g., *lack of recognition for extra work bothers me*), classroom management (e.g., *I find it difficult to discipline the class*), initiative and creativity (e.g., *I try new pedagogical approaches*), subjective work demands (e.g., *I feel I am not doing enough for the students*), working conditions (e.g., *the working conditions I work under are inadequate*), relationships with management (e.g., *I have the opportunity to be actively involved in decisions about the school*), sense of control (e.g., *my work tasks are clear*), personal responsibility (e.g., *I have high expectations of my work*), and relationships with colleagues (e.g., *I do not have many conflicts with my colleagues*). In the second step, we used Varimax with Kaiser normalization as a rotation method. Thirteen components explained 60.1% of the total variance. Difficulty indices of all items were appropriate (0.40 ≤ *p* ≤ 0.89), discrimination index analysis revealed 13 items with an index below 0.20 and 5 items below 0.25. Internal consistency was estimated with Cronbach’s coefficient alpha. Reliability coefficients for the subscales were as follows: 0.72, 0.73, 0.75, 0.75, 0.54, 0.60, 0.68, 0.39, 0.50, 0.47, 0.34, 0.22, and 0.34, respectively.

### 2.3. Data analyses

Data analyses were performed using SPSS 26.0, and graphical representations were created using MS Excel. All statistical analyses, including burnout, were performed with the panel group (*N* = 163). Descriptive and correlational analyses of background variables and environmental and individual factors were performed with the total sample at T1 (*N* = 614).

For attrition analysis, we used the following calculation: $\frac{2\times y\times 100\;}{2\;x+z}$, where *x* is the number of participants at baseline, *y* is the number of participants who left the study at a given time point, and *z* is the number of participants who entered the study at a given time point. In the correlation analysis between the background variables and the burnout index, we used the Eta coefficient, Spearman’s *ρ*, or Pearson’s *r*, depending on whether the variable was nominal, ordinal, or at least interval scaled.

To track the development of burnout over the school year, we calculated the *M*, *SE*, *SD*, and percentages of the burnout dimensions and SFB. Normality of the distributions for all variables was tested using the Kolmogorov–Smirnov test (K–S test). To test for differences between measurements, we used One-way Repeated-Measures ANOVA for EE and PA (*post-hoc* test: *t*-test) and the Friedman test (*post-hoc* test: Wilcoxson signed rank test) for DP and SFB, depending on the results of the K-S test. Correlations between MBI burnout dimensions and SFB were calculated using the Spearman correlation coefficient.

We calculated the M and SD for individual and environmental factors. The normality of the distributions of the categories of the factors was tested using the K-S test. The analysis of the burnout factors was performed in two ways: (1) a rank analysis of the individual and environmental factors according to the teachers’ subjective importance for burnout and (2) a correlation analysis between the categories of the burnout factors and the burnout index.

To examine the relationships between burnout and the categories of environmental and individual factors, we calculated Spearman’s correlation coefficient. In the correlation analysis, we used the burnout index (IB), which ensures the continuity of the index (Demšar, 2003). Formula for calculating *I_B_*: $I_{B}=4\times \frac{M_{\mathrm{EE}}}{6}+2\times \left(\frac{M_{PA}}{6}\right)+1\times \frac{M_{DP}}{6}$. *M_EE_*, *M_PA_*, and *M_DP_* are the average score of the individual on all three dimensions of burnout. The possible range of *I_B_* is from 0 to 6, with a higher score indicating a higher level of burnout.

To examine the predictive contribution of the environmental factor and individual burnout factor categories, multiple regression was conducted using a model *Forward*. The burnout factor category was included in the multiple regression analysis if it was significantly correlated with the burnout index at least two time points. There were nine categories of environmental and individual burnout factors that met this criterion (*time and energy demands of working with students, teacher characteristics, classroom management, student learning characteristics, subjective work demands, administration and job responsibilities, working conditions, ambition*, and *sense of control*).

## 3. Results

### 3.1. Attrition analysis

Attrition analysis showed an attrition rate of 56.4% from T1 to T2 and 34.7% from T2 to T3; the overall attrition rate (from T1 to T3) was 62.3%. To examine whether the leavers differed from the panel group, we compared their background variables (age, gender, teaching experience, urban/rural school district, being a classroom teacher, permanent/fixed-term employment) and dependent variables (average scores for the dimensions of burnout at T1 and 13 environmental and individual factors). Results showed that there were significant differences between groups for type of employment (*χ^2^* = 7.181, *df* = 2, *p* = 0.028), type of school district (*χ^2^* = 35.625, *df* = 2, *p* = 0.000), and being a classroom teacher (*χ^2^* = 11.066, *df* = 2, *p* = 0.026). There were no significant differences between groups on burnout dimensions or environmental and individual factors.

We also calculated correlations between background variables and the burnout index in all three measurements (Table 2).

**Table 2:** Correlation analysis of background variables and burnout index in three time points.

Background variable		Index of burnout in T1	Index of burnout in T2	Index of burnout in T3
Gender	*Eta* Coefficient	0.025	0.004	0.098
	*N*	614	306	321
Age	*r*	**0.163** *	**0.209** *	**0.123** *
	*p*	0.000	0.000	0.028
	*N*	613	306	319
Type of employment	*Eta* Coefficient	0.188	0.180	0.083
	*N*	612	305	320
Years of teaching	*r*	**0.157** *	**0.208** *	**0.113** *
	*p*	0.000	0.000	0.044
	*N*	613	306	319
School district	*Eta* Coefficient	0.009	0.115	0.027
	*N*	614	306	321
Size of the school	*ρ*	0.039	0.075	0.041
	*p*	0.332	0.191	0.462
	*N*	614	306	321
Instructing educational level	*Eta* Coefficient	0.126	0.130	0.138
	*N*	613	306	321
Being a classroom teacher	*Eta* Coefficient	0.024	0.108	0.094
	*N*	612	306	321
Average number of students in the classroom	*r*	0.046	**0.127** *	**0.129** *
	*p*	0.262	0.028	0.023
	*N*	594	302	313

* Correlation is significant at the 0.05 level. Significant correlations are in bold.

All correlations between the background variables and the burnout index are very weak and positive. The burnout index correlates significantly with age, years of teaching experience, and average number of students in the classroom. Since the differences between the sample in the T1 group and the panel group were found in terms of type of employment, type of school district, and being a classroom teacher or not, and these background variables are also not correlated with the burnout index, we can proceed with the statistical analyses, but still with caution in interpretation.

### 3.2. Burnout development

Table 3 shows the descriptive statistics of the burnout questionnaire for the panel group at all three time points. Scores in the upper third of the distributions of EE and DP and in the lower third of PA represent high burnout (Maslach et al., 1996). Accordingly, scores from EE represent moderate burnout and scores from DP and PA represent low burnout for participants over the course of the school year.

**Table 3:** Descriptive statistics of burnout questionnaire for panel group in three time points.

	*N*	Min	Max	*M*	*SD*	Skewness	Kurtosis	Kolmogorov–Smirnov *Z*	*p*
EE/T1	163	3	45	21.02	8.62	0.21	−0.38	0.690	0.728
EE/T2	163	0	50	20.42	9.94	0.20	−0.32	0.783	0.573
EE/T3	163	0	41	19.93	9.90	0.23	−0.70	0.827	0.500
DP/T1	163	0	21	5.65	4.41	0.82	−0.01	2.058	0.000
DP/T2	163	0	21	5.64	4.37	0.81	0.35	1.563	0.015
DP/T3	163	0	18	5.58	4.44	0.69	−0.21	1.356	0.051
PA/T1	163	17	46	33.85	6.00	−0.43	−0.19	1.119	0.163
PA/T2	163	22	48	34.86	5.42	−0.23	−0.23	1.058	0.213
PA/T3	163	11	48	34.19	6.58	−0.39	0.23	0.727	0.666
SFB/T1	163	1	5	2.51	1.03	0.04	−0.87	2.607	0.000
SFB/T2	163	1	5	2.40	0.99	0.05	−0.86	2.781	0.000
SFB/T3	163	1	5	2.75	1.03	−0.16	−0.66	2.913	0.000

**Figure 1:**
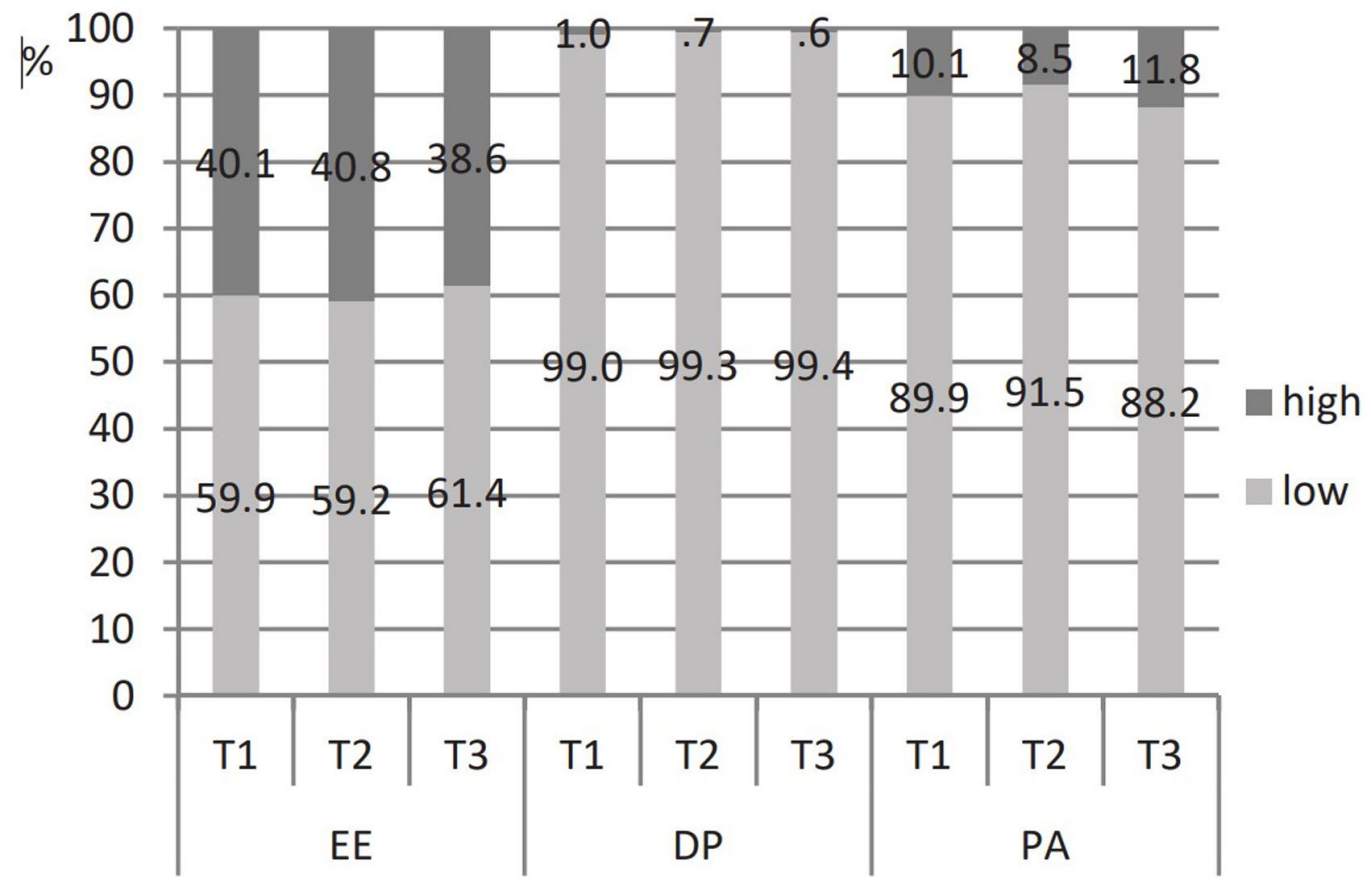
Percentage of teachers with high and low burnout dimensions at three time points.

The percentage of teachers with high burnout on three different dimensions (Figure 1) shows that EE is the most pronounced dimension (about 40% of teachers at all three time points). Low PA is reported by about 10% of teachers, while high DP is reported by no more than 1% of teachers.

Mean scores for the three burnout dimensions vary only slightly over the school year (Table 3). EE and DP decrease slowly over the school year; PA increases from T1 to T2 and decreases in T3, but not below the T1 score. The differences are not statistically significant (*F_EE_* = 1.634, *df* = 2, *p* = 0.197; *χ^2^_DP_* = 0.289, *df* = 2, *p* = 0.865; *F_PA_* = 2.276, *df* = 1.9, *p* = 0.108), except between the values of PA at T1 and T2 (*t* = −2.355, *df* = 162, *p* = 0.020).

**Figure 2:**
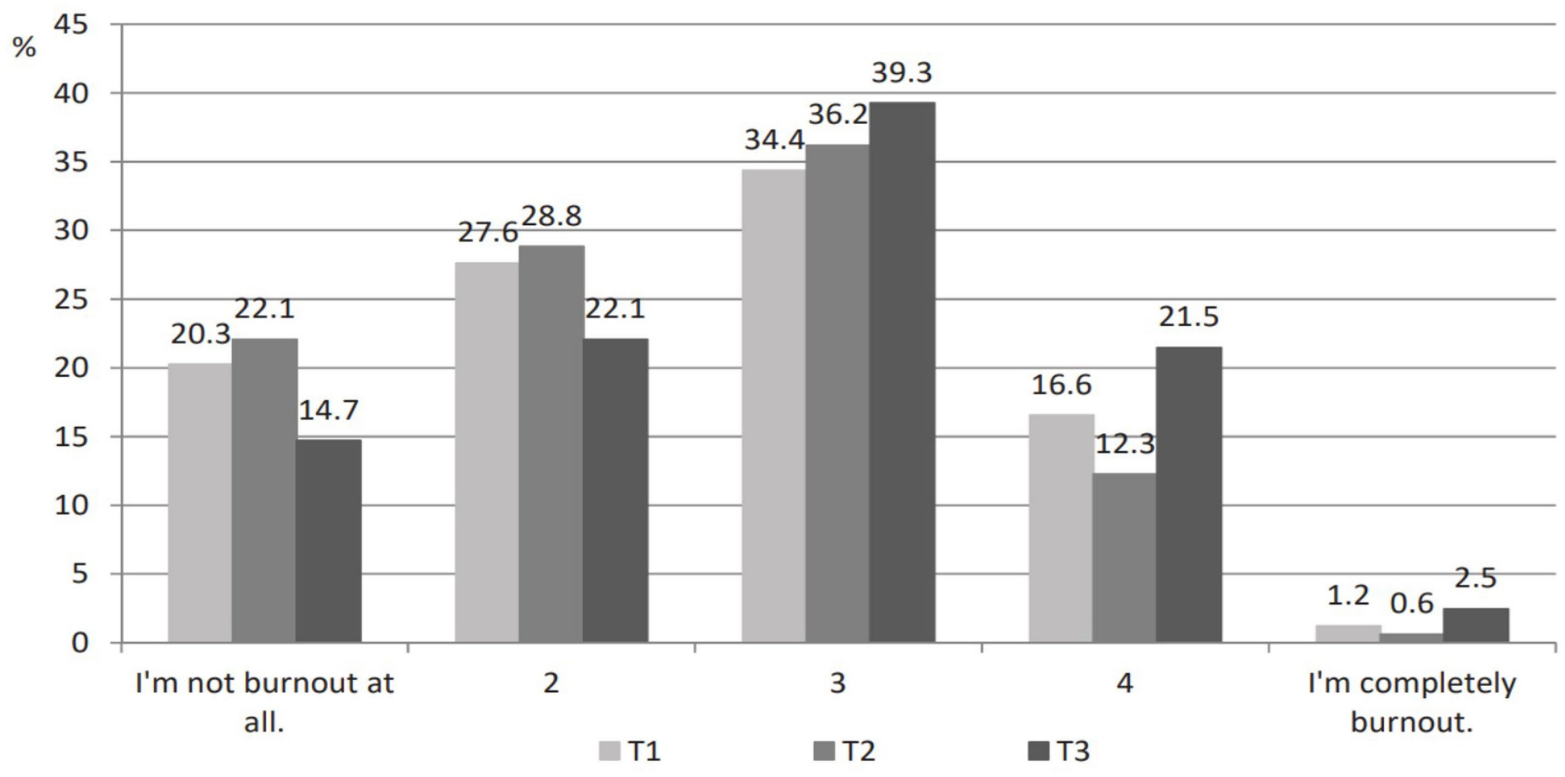
SFB in three time points—percentage of teachers.

Teachers rated their SFB as low to moderate over the year, similar to MBI scores. The mean score of SFB first decreased from T1 (*M* = 2.51, *SD* = 1.03) to T2 (*M* = 2.40, *SD* = 0.99) and then increased from T2 to T3 (*M* = 2.75, *SD* = 1.03). Differences between measurements during the school year were statistically significant (*χ^2^_SFB_* = 16.055, *df* = 2, *p* < 0.001). Post-hoc tests revealed that the difference between T1 and T2 and T1 and T3 was not statistically significant (*Z_1-2_* = −1.509, *p* = 0.131; *Z_1-3_* = −1.757, *p* = 0.079), while the difference between T2 and T3 was statistically significant (*Z_2-3_* = −4.573, *p* < 0.001). Figure 2 shows that at T1 and T2, about 15% of teachers have a strong sense of burnout (scores 4 and 5) and about half have a low or no sense of burnout (scores 1 and 2). At T3, the percentage of teachers decreases significantly for low scores (scores 1 and 2) and increases for higher scores (score 4). At the end of the school year, about a quarter of them have a strong sense of burnout.

Table 4 shows the correlation analysis between MBI dimensions and SFB. There are statistically significant correlations between the dimensions of burnout and SFB for all three measurements, except SFB and PA for the first measurement. The PA dimension is negatively related to the other dimensions of burnout and SFB for all three measurements: the relationship is insignificant to small (−0.35 < *ρ* < −0.10) for the first two measurements and moderate (−0.40 < *ρ* ≤ −0.49) for the third measurement. The correlations between EE and DP are moderately positive for all three measurements (*ρ* about 0.50). SFB is highly positively correlated with EE for all three measurements (0.74 < *ρ* < 0.79) and moderately positively correlated with DP (0.31 < *ρ* < 0.43).

**Table 4:** Correlation analysis of burnout dimensions and SFB for panel group in three time points.

	SFB	EE	DP	PA
T1				
SFB				
*ρ*	1.000			
*p*	.			
*N*	163			
EE				
*ρ*	**0.744** **	1.000		
*p*	0.000	.		
*N*	163	163		
DP				
*ρ*	**0.401** **	**0.508** **	1.000	
*p*	0.000	0.000	.	
*N*	163	163	163	
PA				
*ρ*	−0.096	**−0.239** **	**−0.347** **	1.000
*p*	0.223	0.002	0.000	.
*N*	163	163	163	163
T2				
SFB				
*ρ*	1.000			
*p*	.			
*N*	163			
EE				
*ρ*	**0.784** **	1.000		
*p*	0.000	.		
*N*	163	163		
DP				
*ρ*	**0.428** **	**0.564** **	1.000	
*p*	0.000	0.000	.	
*N*	163	163	163	
PA				
*ρ*	**−0.264** **	**−0.290** **	**−0.328** **	1.000
*p*	0.001	0.000	0.000	.
*N*	163	163	163	163
T3				
SFB				
*ρ*	1.000			
*p*	.			
*N*	163			
EE				
*ρ*	**0.780** **	1.000		
*p*	0.000	.		
*N*	163	163		
DP				
*ρ*	**0.316** **	**0.457** **	1.000	
*p*	0.000	0.000	.	
*N*	163	163	163	
PA				
*ρ*	**−0.395** **	**−0.475** **	**−0.490** **	1.000
*p*	0.000	0.000	0.000	.
*N*	163	163	163	163

** Correlation is significant at the 0.01 level. Significant correlations are in bold.

### 3.3. Burnout levels in relation to teacher demographic characteristics and teaching contexts

The age and years of teaching experience of the participating primary teachers correlate with the burnout index on all three measurements (Table 2). The correlations are weak and positive (0.12 < *r_age_* < 0.21; 0.11 < *r_years of teaching_* < 0.21). There were no statistically significant differences in burnout levels related to gender (Table 5).

**Table 5:** Descriptive statistics and averages of burnout index comparison between gender at three time points.

Gender		*N*	*M*	*SD*	Min	Max	*t*	*p*
Index of burnout—T1	Women	18	2.19	0.78	0.91	3.66	0.567	0.452
	Men	144	2.35	0.84	0.39	4.71		
	All	162	2.33	0.83	0.39	4.71		
Index of burnout—T2	Women	18	1.99	0.93	0.00	3.40	1.638	0.202
	Men	144	2.29	0.93	0.34	5.13		
	All	162	2.26	0.93	0.00	5.13		
Index of burnout—T3	Women	18	2.14	0.90	0.67	4.18	0.227	0.634
	Men	144	2.26	0.98	0.34	4.39		
	All	162	2.25	0.97	0.34	4.39		

The majority of teachers (82.4%) are tenured. Table 6 shows the relationships between the type of employment and the burnout index.

**Table 6:** Descriptive statistics and averages of burnout index comparison between teachers with permanent and temporary position at three time points.

Type of employment		*N*	*M*	*SD*	Min	Max	*t*	*p*
Index of burnout—T1	Temporary	23	1.89	0.69	23	1.89	8.268	0.005*
	Permanent	138	2.41	0.83	138	2.41		
	All	161	2.34	0.83	161	2.34		
Index of burnout—T2	Temporary	23	1.81	1.10	23	1.81	6.583	0.011*
	Permanent	138	2.34	0.88	138	2.34		
	All	161	2.26	0.93	161	2.26		
Index of burnout—T3	Temporary	23	2.09	1.21	23	2.09	0.708	0.401
	Permanent	138	2.28	0.93	138	2.28		
	All	161	2.25	0.97	161	2.25		

* Correlation is significant at the 0.05 level.

The type of employment has been shown to be an important factor related to burnout. Teachers who are employed for a period of time have, on average, lower levels of burnout than tenured teachers. The differences between the groups were also found to be statistically significant at T1 and T2. Teachers with permanent positions have, on average, longer work experience and are also older. They are also more emotionally involved in their work (*F_EE-T1_* = 9.352, *df* = 1, *p* = 0.003; *F_EE-T2_* = 6.360, *df* = 1, *p* = 0.013) than temporary teachers. The latter are likely to be aware that they will leave at the end of the school year and that they have no significant influence on what happens in their current workplace. Consequently, they are more likely to accept the work environment as it is and are less concerned about the current situation.

More than half of the teachers (57.5%) also have classroom teaching responsibilities. Table 7 shows differences in burnout index between teachers who take on a classroom task and teachers who do not at three time points.

**Table 7:** Descriptive statistics and averages of burnout index comparison between teachers who take on a classroom task and teachers who do not at three time points.

Classroom teaching responsibilities		*N*	*M*	*SD*	Min	Max	*t*	*p*
Index of burnout—T1	Yes	98	2.30	0.85	0.39	4.40	0.331	0.566
	No	64	2.38	0.81	0.49	4.71		
	All	162	2.33	0.83	0.39	4.71		
Index of burnout—T2	Yes	98	2.23	0.87	0.34	4.54	0.243	0.623
	No	64	2.30	1.02	0.00	5.13		
	All	162	2.26	0.93	0.00	5.13		
Index of burnout—T3	Yes	98	2.28	0.98	0.45	4.32	0.235	0.629
	No	64	2.20	0.95	0.34	4.39		
	All	162	2.25	0.97	0.34	4.39		

Performing the function of a classroom teacher did not prove to be an important factor in relation to experiencing burnout. In T1 and T3, classroom teachers had higher average burnout indices than non-classroom teachers; in T2, the opposite was true. At none of the measurements did the differences between classroom teachers and non-classroom teachers prove statistically significant. These results are somewhat surprising given that many teachers perceive classroom teaching as an additional and demanding burden. In addition to the greater administrative burden, teachers also perceive classroom teaching as stressful because they subjectively take responsibility for student success, behavior, and experiences.

Depending on educational level (Table 8), teachers teaching at the grade level had the lowest burnout levels, whereas teachers teaching at the subject level or teachers teaching both levels had similar burnout levels at all three time points. We found statistically significant differences in the burnout index only at T1.

**Table 8:** Descriptive statistics and average comparison of burnout index between teachers at different educational level at three time points.

Educational level		*N*	*M*	*SD*	Min	Max	*F*	*p*
Index of burnout—T1	Grade level	70	2.14	0.79	0.39	4.40	4.326	0.015*
	Subject level	55	2.52	0.95	0.55	4.71		
	Teaching at both levels	29	2.52	0.54	1.27	3.39		
	All	154	2.35	0.83	0.39	4.71		
Index of burnout—T2	Grade level	70	2.09	0.89	0.34	4.02	2.797	0.064
	Subject level	55	2.38	1.06	0.00	5.13		
	Teaching at both levels	29	2.52	0.72	1.18	4.54		
	All	154	2.27	0.93	0.00	5.13		
Index of burnout—T3	Grade level	70	2.10	0.93	0.40	4.39	1.509	0.224
	Subject level	55	2.39	1.10	0.34	4.34		
	Teaching at both levels	29	2.33	0.84	0.69	4.01		
	All	154	2.25	0.98	0.34	4.39		

* Correlation is significant at the 0.05 level.

### 3.4. Environmental and individual factors of burnout

Teachers reported an average of 13.5 (*SD* = 4.9) individual factors and factors in the school environment that strongly influence them. Correlation analysis revealed significant positive low to moderately high correlations between the number of burnout factors teachers rated as important and the three burnout dimensions (*ρ* = 0.44 at T1, *ρ* = 0.38 at T2, and *ρ* = 0.32 at T3).

Teachers rated how much they were affected by environmental and individual factors related to burnout. They rated most of the items as averagely burdensome (1.67 ≤ *M_i_* ≤ 4.18). The most stressful items represent work not directly related to teaching (*administrative work*, *complexity of work*, *teachers’ own expectations of their job performance*, and *introduction of new teaching methods*). Environmental and individual burnout factors related to *sense of control*, *relationships with colleagues*, and *time and energy demands of working with students* were rated as least stressful. All items related to teaching and other work with students were in the middle of the scale of burnout predictors according to the ranking of mean scores.

As described in the Method section, PCA analysis yielded 13 categories of environmental and individual predictors of burnout (Table 9). Similar to the item analysis, we can see that the most stressful categories of predictors of burnout among teachers are related to administration and job responsibilities, followed by subjective perception of responsibility. The least stressful categories of predictors of burnout are also similar to the item analysis: *working conditions*, *sense of control*, and *relationships with colleagues* have the least impact according to teachers. The categories of predictors related to students are in the middle of the rankings. Thus, similar to the item analysis, we can conclude that (1) teachers are mainly burdened by work that is not directly related to teaching in the classroom and by their own expectations of work performance, and (2) schools, on average, are likely to take good care of teachers’ working conditions.

**Table 9:** Descriptive statistics of categories of predictors of burnout in T1.

Categories of burnout factors	*N*	Min	Max	*M*	*SD*	Skewness	Kurtosis	Kolmogorov–Smirnov *Z*	*p*
Administration and job responsibilities	610	1.0	5.0	3.85	0.89	−0.69	0.02	2.96	0.000
Personal responsibility	611	1.5	5.0	3.68	0.89	−0.08	−0.96	3.64	0.000
Initiative and creativity	610	1.0	5.0	3.66	0.72	−0.22	0.10	2.27	0.000
Subjective work demands	610	1.0	5.0	3.28	0.65	−0.13	0.18	2.63	0.000
Time and energy demands of working with students	611	1.0	5.0	3.16	0.76	−0.13	−0.34	1.53	0.020
Ambition	610	1.0	5.0	3.07	0.76	0.03	−0.26	1.85	0.000
Classroom management	611	1.0	5.0	2.98	0.76	−0.02	−0.02	2.02	0.000
Relationships with management	610	1.0	5.0	2.71	0.75	0.11	−0.06	2.37	0.000
Student learning characteristics	610	1.0	5.0	2.65	0.81	0.05	−0.29	2.35	0.000
Teacher characteristics	610	1.0	4.8	2.14	0.62	0.52	0.58	2.24	0.000
Working conditions	611	1.0	5.0	2.06	0.93	0.63	−0.31	3.85	0.000
Relationships with colleagues	611	1.0	5.0	1.97	0.93	0.70	−0.42	4.91	0.000
Sense of control	610	1.0	5.0	1.76	0.74	1.08	1.37	4.66	0.000

Correlation analysis of categories of burnout factors (Table 10) showed that burnout was statistically significantly correlated with *time and energy demands of working with students* (0.33 < *ρ* < 0.51), *teacher characteristics* (0.26 < *ρ* < 0.47), *classroom management* (0.26 < *ρ* < 0.40), *student learning characteristics* (0.29 < *ρ* < 0.36), *personal responsibility* (0.23 < *ρ* ≤ 0.34), and *working conditions* (0.15 < *ρ* < 0.24) at all three time points. All correlations were positive and ranged from insignificant to moderate.

**Table 10:** Bivariate correlation between burnout index and categories of factors of burnout in three time points.

Categories of burnout factors		Index of burnout		
		T1	T2	T3
Teacher characteristics	*ρ*	**0.468**	**0.264**	**0.331**
	*p*	0.000	0.000	0.000
	*N*	610	252	241
Time and energy demands of working with students	*ρ*	**0.504**	**0.455**	**0.334**
	*p*	0.000	0.000	0.000
	*N*	611	252	241
Student learning characteristics	*ρ*	**0.359**	**0.297**	**0.327**
	*p*	0.000	0.000	0.000
	*N*	610	252	241
Administration and job responsibilities	*ρ*	**0.219**	**0.214**	0.109
	*p*	0.000	0.001	0.091
	*N*	610	252	241
Ambition	*ρ*	**0.244**	**0.208**	0.097
	*p*	0.000	0.001	0.135
	*N*	610	252	241
Classroom management	*ρ*	**0.392**	**0.378**	**0.265**
	*p*	0.000	0.000	0.000
	*N*	611	252	241
Initiative and creativity	*ρ*	−0.046	0.057	0.044
	*p*	0.255	0.365	0.496
	*N*	610	252	241
Personal responsibility	*ρ*	**0.320**	**0.340**	**0.232**
	*p*	0.000	0.000	0.000
	*N*	610	252	241
Working conditions	*ρ*	**0.231**	**0.155**	**0.167**
	*p*	0.000	0.014	0.010
	*N*	611	252	241
Relationships with management	*ρ*	**0.142**	0.019	0.100
	*p*	0.000	0.763	0.121
	*N*	610	252	241
Sense of control	*ρ*	**0.156**	**0.148**	0.136
	*p*	0.000	0.018	0.035
	*N*	610	252	241
Subjective work demands	*ρ*	−0.006	−0.023	0.079
	*p*	0.883	0.717	0.224
	*N*	611	252	241
Relationships with colleagues	*ρ*	**0.138**	0.093	0.075
	*p*	0.001	0.143	0.247
	*N*	611	252	241

Significance level is set at 0.05. Significant correlations are in bold.

### 3.5. Predictors of burnout

We performed multiple regression separately for three measurements of burnout (Tables 11, 12). The proposed models of predictors for all time points are different yet similar.

**Table 11:** Model summary for multiple regression of burnout predictors with Forward model.

Model summary	*R*	*R^2^*	Adjusted *R^2^*	*SE* of estimate	Change statistics				
					*R^2^* change	*F* change	*df1*	*df2*	*p*
T1	0.694	0.482	0.476	0.665	0.007	8.024	1	602	0.005
T2	0.540	0.292	0.280	0.803	0.027	9.535	1	247	0.002
T3	0.482	0.232	0.219	0.845	0.030	9.204	1	236	0.003

**Table 12:** Coefficients of categories of individual and environmental factors of burnout, model *Forward*.

Measurement	Model	Unstandardized coefficients		Standardized coefficients	*t*	*p*
		*B*	*SE*	*β*		
T1	(Constant)	−1.645	0.194		−8.460	0.000
	Time and energy demands of working with students	0.311	0.046	0.257	6.751	0.000
	Teacher characteristics	0.577	0.047	0.388	12.366	0.000
	Classroom management	0.125	0.043	0.103	2.901	0.004
	Student learning characteristics	0.092	0.038	0.081	2.414	0.016
	Personal responsibility	0.190	0.048	0.135	3.993	0.000
	Administration and job responsibilities	0.098	0.034	0.095	2.867	0.004
	Working conditions	0.086	0.030	0.087	2.833	0.005
T2	(Constant)	−0.898	0.343		−2.621	0.009
	Time and energy demands of working with students	0.341	0.085	0.267	4.030	0.000
	Teacher characteristics	0.285	0.086	0.182	3.325	0.001
	Classroom management	0.199	0.079	0.162	2.502	0.013
	Personal responsibility	0.276	0.089	0.186	3.088	0.002
T3	(Constant)	−0.426	0.332		−1.282	0.201
	Time and energy demands of working with students	0.249	0.088	0.188	2.844	0.005
	Teacher characteristics	0.376	0.093	0.235	4.025	0.000
	Classroom management	0.152	0.086	0.114	1.762	0.079
	Student learning characteristics	0.239	0.079	0.190	3.034	0.003

The model for T1 yielded seven important predictors of burnout that predicted 48.2% of the variance in the burnout index and was statistically significant (*F*(7.602) = 79.97, *p* = 0.000). All predictors were found to be significant. The strongest predictors were *teacher characteristics* (*β* = 0.388) and *time and energy demands of working with students* (*β* = 0.257).

The model for T2 revealed four significant predictors of burnout that predicted 29.2% of the variance in the burnout index and was statistically significant (*F*(4.247) = 25.44, *p* = 0.000). All predictors were found to be significant. The category *time and energy demands of working with students* (*β* = 0.267) also proved to be the strongest predictor of burnout index in this model, followed by the *personal responsibility* (*β* = 0.186) and *teacher characteristics* (*β* = 0.182).

The model for T3 revealed four significant predictors of burnout that predicted 23.2% of the variance in the burnout index and was statistically important (*F*(4.236) = 17.82, *p* = 0.000). All predictors, except *classroom management*, were found to be significant. The category *teacher characteristics* (*β* = 0.235) proved to be the strongest predictor of the burnout index in the final model, followed by *student learning characteristics* (*β* = 0.190) and *time and energy demands of working with students* (*β* = 0.188).

## 4. Discussion

The present study had two objectives. The first was to examine the dynamics of burnout during the school year, and the second was to examine the relationships between burnout and individual and environmental factors in the school context based on a three-wave panel design. Our goal was to examine which individual and environmental factors best predict current and future levels of burnout.

Maslach et al. (1996) claim that scores in the upper third of the distributions of EE and DP and in the lower third of PA represent high burnout. Accordingly, the results of this study indicate that burnout is present, although not pronounced, among participating primary school teachers. Scores from EE represent moderate burnout and scores from DP and PA represent low burnout for participants over the course of the school year. On the other hand, the percentage of teachers experiencing high EE is high and the mean scores of subjective feelings of burnout are higher than the objectively measured level of burnout. Maslach et al. (2001) assert that EE is the most obvious manifestation of burnout. These findings may point to the problem of excessive emotional strain on teachers on the one hand and a lack of support in the workplace on the other. If emotional stress continues, emotional and energetic reserves may decrease in the future and feelings of frustration, anger, hostility, anxiety, and fear may increase. This is consistent with suggestion of Llorens-Gumbau and Salanova-Soria (2014) that the development of burnout in teachers may begin with chronic job demands or stressors that first influence the rise of EE. Interventions for emotionally exhausted teachers are necessary to prevent depersonalization and maintain existing interpersonal relationships and feelings of personal accomplishment.

Correlation analysis between MBI dimensions revealed low negative correlations of PA with the other two dimensions and moderate positive correlations between EE and DP. The correlations between dimensions should be low because the authors of the MBI questionnaire (Maslach et al., 1996) used principal factoring with iteration and orthogonal (varimax) rotation in extracting the factors (dimensions of burnout). Lourel and Gueguen (2007) used a meta-analysis of research on the theoretical dimensionality of MBI to show that DP and PA are always negatively correlated, EE and DP are always positively correlated, and that EE and PA are not always negatively correlated. Maslach et al. (1996) state that EE is the burnout dimension that is most similar to an orthodox stress variable and therefore yields similar results. However, they caution that limiting the concept of burnout to EE means that it is simply defined as experienced stress. Since SFB directly expresses teachers’ experience of burnout, this is the logical reason why SFB correlates so strongly with EE.

Analysis of burnout dynamics over the course of a school year is interesting. In T2, the average scores of PA increased significantly. The explanation could be that after the first assessment period (T2 was about 3 weeks after the end of the first assessment period), teachers saw the first results of their efforts and the feeling of PA increased accordingly. At the end of the school year, a non-significant decrease in PA was observed. Correlation analyzes revealed that the more teachers perceived EE, the more they felt DP. In such a state, teachers certainly feel less PA about their work. Nevertheless, the pathways of burnout development should be further investigated.

Another significant difference that is difficult to explain is the change in subjective feelings of burnout at the end of the school year: On average, teachers experience a stronger sense of burnout than during the rest of the school year. We cannot relate this change to any other change at the end of the school year because there is no other significant change in the measured burnout dimensions. The scores of EE and DP are fairly stable over the course of the school year, but there is a non-significant decrease in PA at the end of the school year. The correlation between SFB and PA increases from insignificant and non-significant at T1 to negatively moderate and significant at T3. A wild guess might be that the subjective feeling of burnout might be related to the stronger correlation between SFB and PA at T3 and a decrease, though not significant, of PA at T3.

Overall, the differences in the three burnout dimensions at the three different time points are mostly not significant, while the increase in teachers’ subjective feelings of burnout at the end of the school year is statistically significant. Our results are consistent with study of Capel (1991) study, which also showed no significant differences in burnout dimensions at the three time points during the year. While in their study most teachers had the highest burnout scores in February, in our study burnout scores were lowest in February. We hypothesize that major events during the school year (e.g., assessment period) contribute to a temporal increase in burnout, particularly the increase in EE. However, we believe that participants were able to reduce the effects of stress through various coping strategies (e.g., Pogere et al., 2019; Herman et al., 2020) and/or replenish their resources (Hobfoll and Shirom, 2000). Furthermore, as stress accumulates over time (Taris et al., 2001), we would expect burnout to increase. However, it appears that the school year is not long enough for burnout to develop. In fact, Hobfoll and Shirom (2000, p. 73) suggest that “the process of burnout is not constant but dynamic and changes over time, and that temporarily eliminating the stress that causes burnout reduces burnout.” We must reject our assumption that teacher burnout increases from the beginning to the end of the school year.

We found a significant relationship between teacher burnout and the number of stressors perceived by teachers in the work context. Teachers who experienced fewer stressors in the school environment and/or rated them as less threatening had lower burnout scores on all three measurements. The correlations found were low to moderately high. According to transactional stress theories, the cognitive appraisal of a stressor in the (work) environment is an important mediating factor between the effects of a potential stressor and the individual’s stress response (Stoeber and Rennert, 2008; Depolli Steiner, 2014). The development of burnout is influenced by the amount of microstressors currently impacting the individual or their overall impact over time (Taris et al., 2001). Accordingly, the findings of this study suggest that the more microstressors teachers experience, the more likely they are to develop burnout over time, depending, of course, on their cognitive appraisal, coping strategies, replenishment of resources, and other mediating factors of burnout development (Kim et al., 2019; Herman et al., 2020). This is of particular interest in the context of data on the high average number of perceived stressors among teachers.

The ranking of stressors by mean scores suggests that (1) teachers are particularly stressed by work not directly related to teaching and by their own performance expectations, and (2) teachers do not perceive objective working conditions as stressful. Therefore, we assume that, on average, schools manage work organization and organizational climate quite well, so that teachers do not perceive these potential stressors as stressful.

Correlation analyses of potential stressors and other burnout factors revealed several significant associations between burnout and potential individual and environmental factors in the school context. These are: (1) *teacher characteristics*, (2) *time and energy demands of working with students*, (3) *student learning characteristics*, (4) *classroom management*, (5) *subjective work demands*, (6) w*orking conditions*, (7) *administration and job responsibility*, (8) *ambition*, and (9) *sense of control*. The most important factors are related to the time and energy teachers invest in their work with students. This is understandable because the time and energy spent directly affect fatigue (Boksem and Tops, 2008; Hockey, 2013). They may also be related to teacher expectations: When teachers invest more energy in classroom management than they expect, they are more emotionally taxed (Gilmour and Wehby, 2020). Consistent with previous research (e.g., Kim et al., 2019), teacher personality traits were also highly associated with burnout, but this needs further investigation.

On the other hand, the comparison of rank and correlation analyses revealed a partial discrepancy between the results. We have already found that the most important factors for teachers primarily include those related to administration and responsibility for work [consistent with previous research of Kokkinos (2007) and Stauffer and Mason (2013)], as well as the subjective feeling of perceiving one’s own responsibility for a job well done [consistent with previous research of Ekstedt and Fagerberg (2005) and Tomic et al., (2004)]. We found that the first factor was significantly related to burnout, but the second was not. Thus, teachers feel that extra work is the main cause of their burnout. In contrast, the correlation analysis showed that the most significant associations with burnout were teachers’ personality characteristics and their commitment to students. This suggests that the subjective ranking of stressors differs in importance from the objectively measured correlation with burnout. Or, to put it another way, teachers perceive some potential stressors in their environment to be very stressful, but at the same time are unaware that some other potential stressors are much more stressful to them than they perceive. Risk factors that teachers are not aware of are their personality traits, the time and energy they spend working with students, and student learning characteristics. On average, teachers do not perceive themselves as a key factor in the process of transforming the occurrence of stressors and the development of burnout. They may not be aware that personal strength, a positive outlook on life, high self-esteem, work motivation, and job satisfaction are important factors in preventing the occurrence and development of burnout. In addition, teachers do not perceive the time and energy they invest in preparing lessons and working with students as stressful-they likely see it as part of the educational work they have chosen to do. Therefore, they do not subjectively perceive these potential factors as stressful. The same is likely true for student learning characteristics, which are also significantly associated with burnout. Presumably, teachers perceive factors indirectly related to pedagogical work, as well as factors they can influence and believe they can change (e.g., the amount of administrative work), as particularly stressful. However, they do not appear to be stressed by conditions they do not believe they can change.

Multivariate regression analyses revealed three similar models of predictors of burnout. Teacher burnout at all three time points was significantly related to *time and energy demands of working with students*, *teacher characteristic*, and *classroom management*. This means that teachers who are less satisfied with their work, less motivated, have lower personal resilience and self-esteem, and have a more negative outlook on life are more likely to experience burnout. Burnout is also more common among teachers who feel they do not have enough time to work individually with students or implement the curriculum, and among teachers for whom classroom management is challenging.

In addition, at the beginning of the school year, teachers who are more emphatic and have high expectations for their own work, teachers who do not perceive working conditions as adequate and believe they have a lot of administrative and responsible work to do, and teachers who find assessment, differentiation, and working with students with low motivation challenging are more likely to experience burnout. After the first assessment period in the middle of the school year, burnout was additionally experienced by teachers who are more emphatic and have high expectations for their own work, and at the end of the year by teachers who estimate that they teach students with lower learning skills, poor knowledge, and poor study habits.

This study found that predictors of burnout depended on the timing of measurement during the school year. It appears that the dynamics of pedagogical work change over the course of the school year, such that different models better predict levels of burnout at different times during the school year. Regardless of the instability of the models of individual and environmental antecedents, they explain 25–50% of the variance in teacher burnout.

In conclusion, the study found that burnout was present but not pronounced among participating teachers: EE is moderately high and DP and AP are low. Over the course of the school year, burnout did not increase consistently and gradually; only PA and SFB increased statistically significantly. The number of stressors perceived by teachers in the workplace was significantly related to burnout rates. Teachers experience stress, particularly in work not directly related to teaching and through their own performance expectations. Multivariate regression analyses revealed three different but similar models of predictors of burnout. Regardless of the instability of the models, *time and energy demands of working with students*, *teacher characteristics*, and *classroom management* are the stable antecedents in the predictor models of teacher burnout.

The results of this study indicate that the school year is not long enough for burnout to develop. This study suggests that the dynamics of stressors in a school year are not strong enough to influence the development of burnout. Gold and Roth (1993) believe that burnout develops gradually when a person is exposed to stress over a long period of time or when an individual’s needs and wants at work are not met over a long period of time and the individual is unable to eliminate the negative consequences of the stress. More attention should be paid to the development of burnout over the course of a teacher’s career.

In addition, the burnout measurements were evenly distributed throughout the school year and were conducted the week before the vacations, as we assumed that teachers were most tired before the vacations and had renewed energy after the vacations. Given the dynamics of school work during the year, we recommend scheduling measurements by assessment period—before the start of the school year, before the end of the first assessment period, and before the second assessment period. It is even better to conduct the measurements before and after the end of each assessment period. The amount and intensity of work is tied to the end of the assessment periods, not the holiday periods.

The major limitation of this study was the changing composition of the sample during the school year. The initial sample was large and representative, but the dropout rate of participating teachers was high on each subsequent measurement. Nevertheless, the attrition analysis showed no significant differences between the initial sample and the panel group on background variables and burnout dimensions or on environmental and individual factors. We did not examine the causes of attrition. We hypothesize that the teachers who dropped out at higher rates were those who already felt overwhelmed and those who did not feel burned out at all and therefore did not find it useful to complete the questionnaire. This may explain why the average burnout rate remained about the same throughout the school year. At the same time, some teachers did not fill out the questionnaires for various reasons only at the first measurement or only at the second measurement.

## Data availability statement

The raw data supporting the conclusions of this article will be made available by the authors, without undue reservation.

## Ethics statement

Ethical review and approval was not required for the study on human participants in accordance with the local legislation and institutional requirements. Written informed consent for participation was not required for this study in accordance with the national legislation and the institutional requirements.

## Author contributions

NM contributed to the conception and design of the study, organized the database, performed the statistical analysis, and wrote the manuscript. All authors contributed to the article and approved the submitted version.

## Conflict of interest

The authors declare that the research was conducted in the absence of any commercial or financial relationships that could be construed as a potential conflict of interest.

## Publisher’s note

All claims expressed in this article are solely those of the authors and do not necessarily represent those of their affiliated organizations, or those of the publisher, the editors and the reviewers. Any product that may be evaluated in this article, or claim that may be made by its manufacturer, is not guaranteed or endorsed by the publisher.
